# Top-Down Control of Herbivory by Birds and Bats in the Canopy of Temperate Broad-Leaved Oaks (*Quercus robur*)

**DOI:** 10.1371/journal.pone.0017857

**Published:** 2011-04-04

**Authors:** Stefan M. Böhm, Konstans Wells, Elisabeth K. V. Kalko

**Affiliations:** 1 Institute of Experimental Ecology, University of Ulm, Ulm, Germany; 2 Biodiversity and Climate Research Centre (BiK-F), Frankfurt, Germany; 3 Smithsonian Tropical Research Institute, Balboa, Panamá; University of Western Ontario, Canada

## Abstract

The intensive foraging of insectivorous birds and bats is well known to reduce the density of arboreal herbivorous arthropods but quantification of collateral leaf damage remains limited for temperate forest canopies.

We conducted exclusion experiments with nets in the crowns of young and mature oaks, *Quercus robur,* in south and central Germany to investigate the extent to which aerial vertebrates reduce herbivory through predation. We repeatedly estimated leaf damage throughout the vegetation period.

Exclusion of birds and bats led to a distinct increase in arthropod herbivory, emphasizing the prominent role of vertebrate predators in controlling arthropods. Leaf damage (e.g., number of holes) differed strongly between sites and was 59% higher in south Germany, where species richness of vertebrate predators and relative oak density were lower compared with our other study site in central Germany. The effects of bird and bat exclusion on herbivory were 19% greater on young than on mature trees in south Germany.

Our results support previous studies that have demonstrated clear effects of insectivorous vertebrates on leaf damage through the control of herbivorous arthropods. Moreover, our comparative approach on quantification of leaf damage highlights the importance of local attributes such as tree age, forest composition and species richness of vertebrate predators for control of arthropod herbivory.

## Introduction

The question of “Why is the world green?” raised by Hairston et al. [1] has encouraged many ecologists to investigate whether herbivore population dynamics are limited by the availability of food plants and plant defence mechanisms or rather by top-down control through predators. In each case, herbivorous arthropods play a decisive role in ecosystem functioning because, as mid-trophic level species, they are influenced by bottom-up and top-down forces. In turn, herbivorous arthropods impact the fitness of many plant species and associated nutritional cycles [2]–[3].

Numerous studies have demonstrated that leaf quality (e.g. content of secondary metabolites) is a major determinant affecting the distribution and abundance of herbivorous arthropods [3]–[6]. In addition, studies focusing on top-down forces have revealed significant impacts of predators on lower trophic levels, such as herbivorous arthropods [7]–[11]. These, in turn, can drastically reduce leaf area through their feeding activities and hence can affect the biomass and fitness of trees [6], [12]–[17].

The function of predators in food webs is assumed to be positively correlated with species richness and abundance. Higher species diversity increases the likelihood of the presence of more specialized and/or efficient predators that will eventually feed on species not consumed by generalist predators and hence increase the overall range of prey species. This, in turn, might also affect functional relationships over multiple levels in food webs such that mid-trophic level species (e.g. herbivorous arthropods) impact primary producers [7], [18]–[19]. However, empirical evidence combined with the quantification of leaf damage as a means to demonstrate the effects of vertebrates as top-down controllers of herbivores is still scarce, especially in the canopies of temperate ecosystems.

Whereas various field studies suggest that abundance of terrestrial arthropods in the understorey (i.e. in shrubs and small trees) is tightly controlled by insectivorous birds and bats in both tropical and temperate forests, plantations and gardens [8]–[11], [14], [20]–[27], only limited empirical evidence for this control is available to date in temperate forest canopies. Among the few exceptions are the studies of Gunnarsson and co-workers reporting the reduction of spruce-living spiders through insectivorous birds in Sweden [28]–[30]. Studies in Europe on the control of herbivory by birds in forest canopies are limited to oaks (*Quercus pyrenaica* [13]) in the Spanish Pyrenees and to apple orchards in The Netherlands [25]. Whereas Mols & Visser [25] have demonstrated that the abundance of *Parus major* (great tit) is negatively correlated with the number of caterpillars feeding on apples, Sanz [13] has shown that the amount of leaf damage and abundance of caterpillars decreases with increasing number of insectivorous birds.

Although comparative studies from different tree species are important to determining whether the impact of insectivorous predators on leaf damage is similar within and between temperate ecosystems *per se*, a next important step is to take environmental heterogeneity and various diversity scenarios across trophic levels into account. Such studies need to compare regions that differ in one or more of the following traits: species richness and abundance, land-use, climatic zones, or climate change [31].

Here, tree crowns of *Quercus robur* (common oak) play particularly important roles in temperate forest food webs as they harbour large numbers and highly diverse assemblages of arthropods [32]–[34]. Although oaks are distributed across central Europe, they vary in local abundance mainly because of differences in forest management practices. Nowadays, in northern and central Europe natural beech and oak forests have been almost completely replaced by tree plantations of commercial interest [35], in particular conifers. Consequently, the abundance of oaks is low across most of its original distribution range. However, given the high economic value of oak wood and the higher resilience of deciduous trees against storm damage compared to conifer monocultures, which have suffered serious damage in the past decades, a rethinking in forest management practice with a stronger focus on deciduous forests has recently taken place. This is an important development also in terms of maintenance of biodiversity and ecosystem services, and may also affect the control of herbivorous insects by vertebrates.

As most bird species in temperate forests feed on herbivorous arthropods, they favour oaks as foraging substrate over other tree species during the vegetation period [36]. It is of particular interest whether the intensive foraging activities of insectivorous birds in oaks impact the abundance of herbivorous arthropods in canopies and the way in which this might occur. Specifically, quantification of the cascading effects of predation on herbivore abundance and the amount of leaf damage might contribute to a better understanding of ecosystem processes on a broader scale. Furthermore, as the diversity of predators is positively correlated with the amount of herbivore reduction, investigations of ecosystem functioning under various diversity scenarios of predators and prey might enhance possibilities for implementing site-specific conservation plans [13], [18].

In this study, we experimentally excluded birds and bats from the tree crowns of oak trees to assess their impact as predators on intensity of arthropod herbivory on *Quercus robur*. We selected two regions in Germany that differ in overall forest composition and oak density as well as in species richness of vertebrate predators. We conducted studies on both young and old trees and quantified the numbers of chewed holes and percentages of missing leaf area caused by arthropod feeding activities. We expected that intensity of arthropod herbivory in the canopy would increase with the exclusion of vertebrate predators. Furthermore, the diversity of vertebrate predators might also influence intensity of arthropod herbivory, with a higher species richness and abundance of foliage-gleaning birds and bats leading to a stronger reduction in leaf damage.

## Methods

### Study area

This study was part of the large-scale project of the research platform Biodiversity Exploratories [37] with study sites on the Schwäbische Alb near the city of Münsingen (south-western Germany; N 48°25′, E 9°26′) and in the Hainich-Dün National Park near Mühlhausen (central Germany; N 51°13′, E 10°27′). The Schwäbische Alb is characterized by submontane calcareous bedrock (500–900 m a.s.l.) with 6–7°C annual mean temperatures and 700–1,000 mm annual mean precipitation. About 41% of the area is covered by forest patches typically consisting of beech, deciduous-mixed and spruce monocultures, whereas oak trees are relatively rare. The Hainich-Dün consists of a limestone area (300–400 m a.s.l.) with an annual mean temperature of 6.5–7.5°C and annual mean precipitation of 750–800 mm. Approximately 24% of the area is covered with forest; the area contains one of the largest forests of Germany with 16,000 hectares of beech and beech-mixed forests that harbour numerous oak trees within stands, (for more details, see Fischer et al. [37]).

### Selection of study trees and set-up of exclosures

We conducted experiments, over two consecutive years, to exclude vertebrate predators from oaks. In 2007, we randomly selected 12 young common oaks (*Quercus robur*, ≈ 15 years old) along a forest track within a 3-ha oak plantation (pole wood) on the Schwäbische Alb. In 2008, we conducted experiments on 16 randomly selected mature trees with eight trees on the Schwäbische Alb and eight trees in the Hainich-Dün (both stands ≈ 80–120 years). On the Schwäbische Alb, oaks were located at the edge of a beech-dominated forest. In Hainich-Dün, the oaks stood within a patch of deciduous-mixed forest. The distance between individual trees ranged from 15 to 1,700 m (mean 171.5±203.7 m) within each exploratory.

Half of the trees were used as controls and half were fitted each with 150-m^2^ bird exclusion nets (mesh size 20×20 mm, polypropylene, material thickness 1–1.5 mm, black, Huck, Asslar-Berghausen, Germany). Nets covered the whole canopy of young trees and about one third of the total crown volume (≈ 1,000 m^3^) of mature oaks. The nets were tied at the stem to prevent birds and bats from entering. The canopy nets of mature trees were installed by professional tree climbers. Trees were covered with nets between July and October 2007 and between June and October 2008 (Table 1).

**Table 1:** Date of leaf sampling and amount of leaf damage in exclusions and controls.

		Schwäbische Alb			Hainich-Dün		
Study trees	Measurement	Sampling date	Exclusion	Control	Sampling date	Exclusion	Control
Young trees	Mean % damage of leaf area	18-Jul-2007	0.60±0.05	0.36±0.03	-	-	-
		29-Aug-2007	0.91±0.05	0.61±0.04	-	-	-
		08-Oct-2007	0.98±0.06	0.54±0.04	-	-	-
	Mean # holes per leaf	18-Jul-2007	0.98±0.06	0.78±0.09	-	-	-
		29-Aug-2007	1.30±0.07	0.90±0.10	-	-	-
		08-Oct-2007	1.52±0.07	1.00±0.10	-	-	-
Mature trees	Mean % damage of leaf area	02-Jun-2008	0.33±0.03	0.33±0.03	13-Jun-2008	0.23±0.03	0.20±0.03
		25-Jul-2008	0.58±0.04	0.52±0.04	05-Aug-2008	0.43±0.04	0.39±0.04
		08-Sep-2008	0.94±0.07	0.55±0.04	19-Sep-2008	0.66±0.06	0.25±0.03
		17-Oct-2008	0.73±0.05	0.69±0.04	22-Oct-2008	0.56±0.05	0.21±0.03
	Mean # holes per leaf	02-Jun-2008	0.76±0.05	0.74±0.05	13-Jun-2008	0.37±0.03	0.29±0.03
		25-Jul-2008	0.97±0.05	0.79±0.05	05-Aug-2008	0.61±0.05	0.58±0.04
		08-Sep-2008	1.29±0.06	0.90±0.05	19-Sep-2008	0.88±0.06	0.44±0.04
		17-Oct-2008	1.00±0.05	0.94±0.05	22-Oct-2008	0.75±0.05	0.29±0.04

Leaf damage in *Quercus robur* from exclusion and control trees given as means of square-root transformed measures of leaf area damage ± SE and mean number of holes ± SE per leaf. Leaf damage was investigated during repeated samplings of young (6 exclusion and 6 control trees) and mature (8 exclusion and 8 control trees per study area) *Quercus robur* in the two regions Schwäbische Alb and Hainich-Dün. At each sampling date, 60 leaves per tree were collected.

### Leaf sampling and phytometric analysis

Leaves of young oaks were repeatedly sampled during the vegetation period between July and October in 2007 (three times: 18-Jul, 29-Aug, and 08-Oct) and leaves from mature trees were collected between June and October in 2008 (four times: 02-Jun, 25-Jul, 08-Sep, and 17-Oct) to assess leaf damage. Each sample consisted of 60 randomly collected leaves per tree. Although this sample comprised only a small proportion of leaves available in a tree crown, we expect this random sample to be sufficient to examining treatment effects on relative herbivory. Our sample size followed other studies about the impact of birds on leaf damage [e.g., 13]. The leaves were flattened with a Perspex plane on millimetre paper and photographed with a digital camera (350D, Canon, Tokyo, Japan).

For each leaf, we calculated leaf damage by differentiating between mean percentage of damaged leaf area and mean number of holes (≥1 mm^2^) per leaf chewed by arthropods. Missing leaf area was measured with ImageJ 1.40 (Wayne Rasband, National Institutes of Health, Bethesda, USA) by using a polygon selection tool. If holes were located at the edge of the leaf, the missing edge was manually reconstructed. The remaining undamaged leaf area was measured with WinRhizo*Pro* (Regent Instruments Inc., Ottawa, Canada) by automatic colour analysis measurements.

### Vertebrate occurrence and species richness at study trees

Birds were monitored near the study trees within a 50-m radius. We conducted five 60-minute surveys between June and October 2007 near young oaks and between May and October 2008 near mature oaks and investigated species richness and abundance of birds on the basis of sightings and acoustic encounters.

In addition, we used bat monitoring data of a large-scale monitoring study on the Schwäbische Alb and in the Hainich-Dün that was conducted in the vicinity of the study trees. Species richness and activity of bats were acoustically recorded by conducting a line-transect monitoring with a bat detector (Pettersson D1000X) and subsequent analysis of the recorded calls in the lab (Kirsten Jung, unpublished data).

### Statistics

We used generalized linear mixed models (GLMM) fitted by the Laplace approximation with an assumed quasipoisson distribution and a *log*-link [38] to test for the effects of vertebrate exclusion on leaf damage (mean damage of leaf area (%) and mean number of holes per leaf) by taking repeated samplings into account. The treatment and time of leaf sampling were used as fixed effects, whereas the randomly selected individual trees were considered as random effects in the models. Models were fitted separately for the two sampling years and regions. To analyse the effects of the study site and the age of the trees on leaf damage, we constructed another GLMM by using treatment, study site, age of trees, and sampling time as fixed effects. Tree individuals were treated again as a random effect in this model. Significance of treatment was tested by a comparison of models fitted with and without treatment with the *anova* command in R 2.10.0 based on model deviances (R Development Core Team 2010), GLMMs were fitted with the package lme4 (version 0.999375–32 [39]). Leaf area damage and number of holes were square-root-transformed for analysis; means are given ± 1 SE. To compare species richness of birds between young trees and mature oaks in the two study sites we used the One-way Anova.

### Ethics statement

This study was carried out in strict accordance with the laws given by the responsible state environmental offices of Baden-Württemberg and Thüringen (permit 55-8/8848.02-07). We did not affect the 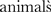 welfare by excluding birds and bats from tree crowns with nets.

## Results

Our study revealed a strong effect of vertebrates on intensity of arthropod herbivory on *Quercus robur* in the two environmental settings tested (young *versus* mature oaks, and mature oaks at the two study sites; all GLMM *anova* model comparisons with/without exclusion treatments P<0.05). The exclusion of vertebrate predators led, in all cases, to a greater mean damage of leaf area (%) and larger mean number of holes per leaf, suggesting that birds and bats significantly reduced the number of leaf-chewing arthropods and thus herbivory (Table 1 and 2).

**Table 2:** Effects of vertebrate exclusion, tree age, and study site on leaf damage.

Analysis	Study site	Study trees	Mean leaf damage	Statistics	Log Likelihood; AIC
Exclusion effect	Schwäbische Alb	Young	% damage of leaf area	χ^2^ = 12.27, df = 1, P<0.001	Model 1: −232.79; AIC = 475.6
					Model 2: −238.92; AIC = 485.9
			# holes per leaf	χ^2^ = 15.11, df = 1, P<0.001	Model 1: −273.41; AIC = 556.8
					Model 2: −280.96; AIC = 569.9
		Mature	% damage of leaf area	χ^2^ = 4.48, df = 1, P = 0.03	Model 1: −608.05; AIC = 1226.1
					Model 2: −610.28; AIC = 1228.6
			# holes per leaf	χ^2^ = 6.14, df = 1, P = 0.01	Model 1: −677.06; AIC = 1364.1
					Model 2: −680.12; AIC = 1368.3
	Hainich-Dün	Mature	% damage of leaf area	χ^2^ = 6.70, df = 1, P = 0.01	Model 1: −592.88; AIC = 1195.8
					Model 2: −596.23; AIC = 1200.5
			# holes per leaf	χ^2^ = 6.08, df = 1, P = 0.01	Model 1: −697.61; AIC = 1405.2
					Model 2: −700.65; AIC = 1409.3
Age effect	Schwäbische Alb	Young/mature	% damage of leaf area	χ^2^ = 5.24, df = 1, P = 0.02	Model 1: −1450.5; AIC = 2913.0
					Model 2: −1453.1; AIC = 2916.3
			# holes per leaf	χ^2^ = 5.81, df = 1, P = 0.02	Model 1: −952.92; AIC = 1917.8
					Model 2: −955.82; AIC = 1921.6
Site effect	Schwäbische Alb/Hainich-Dün	Mature	% damage of leaf area	χ^2^ = 10.92, df = 1, P<0.001	Model 1: −1207.3; AIC = 2426.5
					Model 2: −1212.7; AIC = 2435.4
			# holes per leaf	χ^2^ = 15.76, df = 1, P<0.001	Model 1: −1380.4; AIC = 2772.7
					Model 2: −1388.2; AIC = 2786.5

Statistical results (generalized linear mixed models with log likelihood estimates and AIC) of the analysis of the effects of vertebrate exclusion, tree age, and study site on leaf damage given as mean damage of leaf area (%) and mean number of holes per leaf. Significance of treatments were tested by model comparison fitted with (Model 1) and without factor “exclusion”, “age”, or “site”, respectively (Model 2) with the *anova* command in R 2.10.0 based on model deviances.

**Figure 1:**
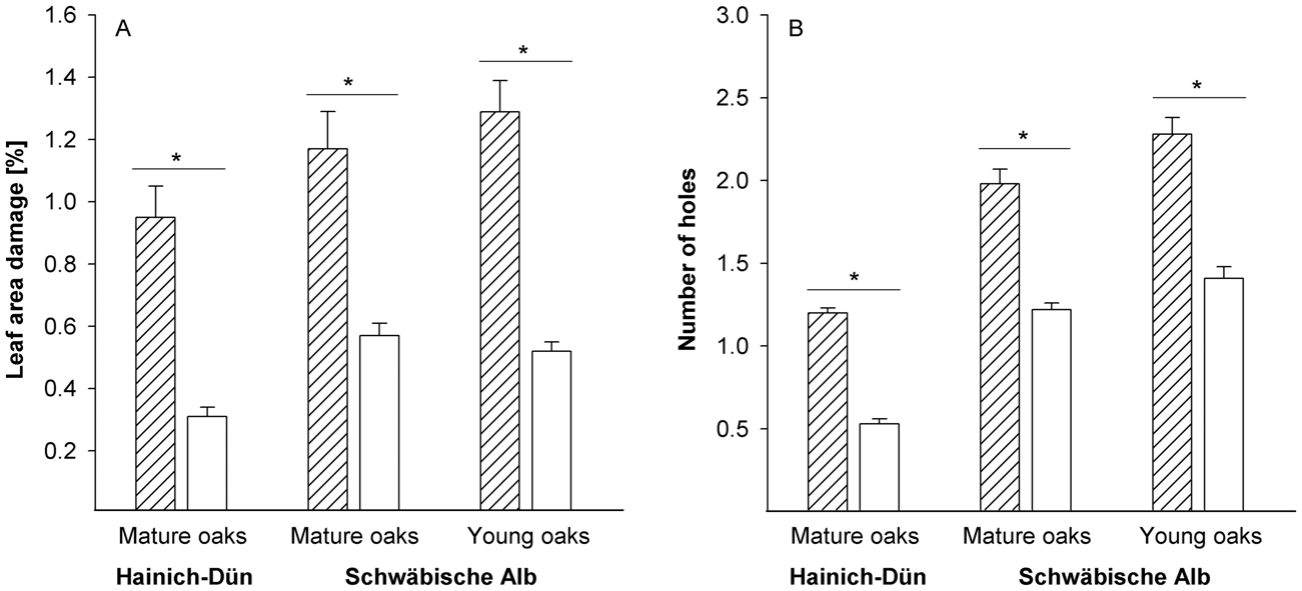
Impact of vertebrate exclusion on leaf damage. Differences in mean damage of leaf area (A) and mean number of holes (B) per leaf for exclusions (shaded bars) and controls (open bars) at the two study sites and for young and mature oaks on the Schwäbische Alb. Significant differences in variables are indicated by “*”. Error bars indicate one SE; variables are presented as square-root transformed values.

On young oaks, mean damage of leaf area (40%, Table 2, Fig. 1A) and mean number of holes per leaf (29% increase, Table 2, Fig. 1B) were significantly higher in netted trees than in controls. Similarly, on mature trees, mean damage of leaf area (Schwäbische Alb 23%, Hainich-Dün 44%, Table 2, Fig. 1A) and mean number of holes per leaf (Schwäbische Alb 16% increase, Hainich-Dün 39% increase, Table 2, Fig. 1B) were significantly greater within the exclusions than on control trees.

**Figure 2:**
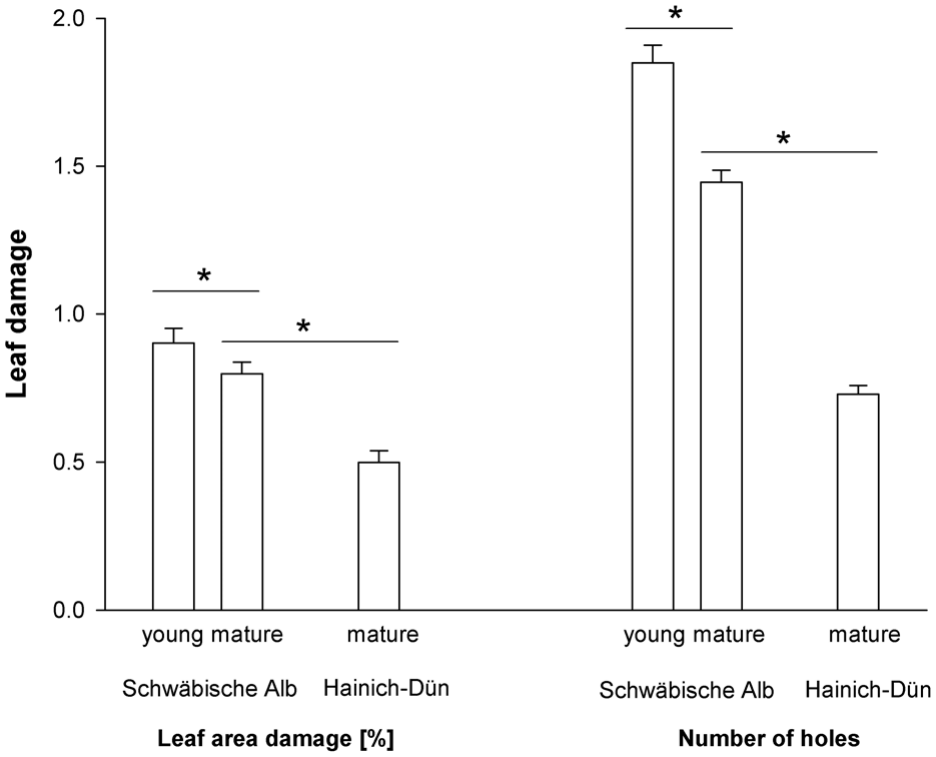
Impact of study region and tree age on leaf damage. Differences in mean damage of leaf area and mean number of holes per leaf between the study regions and young and mature oaks on the Schwäbische Alb. Significant differences in variables are indicated by “*”. Error bars indicate one SE; variables are presented as square-root transformed values.

The extent of leaf damage differed between young and mature oak trees and between the two study regions. In mature trees, the mean damage of leaf area (34%, Table 2) and mean number of holes per leaf (45% increase, Table 2, Fig. 2) were significantly higher on the Schwäbische Alb than in Hainich-Dün. Furthermore, the damage of leaf area (19%, Table 2) and mean number of holes per leaf (22% increase, Table 2, Fig. 2) were significantly higher in young compared with mature trees on the Schwäbische Alb.

The number of observed bird species in the vicinity of the study trees differed between young and mature oaks on the Schwäbische Alb and among the regions. We recorded most species (N = 22) and individuals (N = 53) in the vicinity of mature trees in the Hainich-Dün (One-way Anova, species: F = 9.61, df = 2, P = 0.004, Tukey P<0.05, individuals: F = 16.7, df = 2, P<0.001, Tukey P<0.05) as opposed to only 14 species (27 individuals) and 11 species (15 individuals) in the vicinity of mature and young trees on the Schwäbische Alb, respectively. Similarly, in the Hainich-Dün, more foliage-gleaning bat species (seven species: *Plecotus auritus*, *P. austriacus*, *Myotis bechsteinii*, *M. nattereri, M. emarginatus, M. mystacinus* and *Eptesicus serotinus*) and an up to seven times higher overall bat activity (passes per minute) were found during our large-scale sampling than on the Schwäbische Alb with only four species (*P. auritus*, *P. austriacus*, *M. nattereri* and *M. mystacinus*; Kirsten Jung, unpublished data).

## Discussion

Empirical evidence of quantitative effects of vertebrate predation on leaf damage is scarce, especially for European temperate forest ecosystems. Whether and in what manner the effects of vertebrate predation on intensity of arthropod herbivory can be quantified and associated with predator diversity, forest composition and tree age are of central importance for a deeper understanding of ecosystem processes and the role of diversity on its function.

In accordance with our expectations, this study convincingly shows that the exclusion of birds and bats from tree crowns of the temperate tree species *Quercus robur* results in significantly higher intensity of arthropod herbivory. This result provides strong evidence that the consumption of herbivorous arthropods by birds and bats represents an important ecosystem service of predators in temperate forest canopies.

Although we could not include an analysis of the type and number of herbivorous arthropods in our work, the distinct increase in herbivory within the exclusions suggests that birds and bats limit arthropod feeding activity by reducing their numbers. We propose that the increase of leaf damage measured as the loss in leaf area and the increase in the mean number of holes per leaf in netted oak trees (young and mature trees) are based on an increase of arthropod abundance. Our estimates of leaf damage are in accordance with other studies on understorey plants (e.g. bilberry stands, coffee, vines, tree saplings) in northern Europe and in north and central America. There, insectivorous birds have been shown to reduce arthropod populations, mainly caterpillars, to about half of their population density and leaf damage to around 50% [12], [20]–[22], [27], [40]. Thus, herbivory control through vertebrate insectivores is undoubtedly an important process affecting leaf damage in temperate forest canopies of both young and mature trees in Europe. The extent of the top-down control, however, differs in relation to the age of trees and the composition of forest stands, which strongly influence predator diversity; increasing forest age and tree species diversity are known to increase bird and bat diversity [41]–[42].

Leaf damage was greater in young relative to mature trees, suggesting that young trees harbour more herbivores than mature oaks, as seen in other tree species [13]–[14]. This pattern might be linked in part to the amount of secondary metabolites produced by the plants. Secondary metabolites such as tannins form part of the chemical defence of a plant against herbivorous arthropods and usually occur at lower concentrations in the leaves of young than in the leaves of mature trees, as they are costly to produce [32], [43]–[45]. Another reason for the higher intensity of arthropod herbivory on young trees might be that the relatively dense tree crowns of young trees provide less favourable foraging grounds for foliage-gleaning birds and bats that are active in the canopy and that prefer more open spaces [36], [46]. These assumptions are in agreement with our observations on the Schwäbische Alb where species richness of foliage-gleaning birds was lower in the vicinity of young oaks compared with mature trees.

We cannot rule out that differences in leaf damage between young and mature trees of the Schwäbische Alb might also be influenced by annual fluctuations in arthropod abundance as we sampled young and mature trees in two different years. We did not find any signs for obvious differences in arthropod abundance between the two years and climatic conditions, for instance, were similar in both years (German Weather Service, pers. com.), although further consideration of possible variation in annual interactions awaits further research.

In accordance with our expectations that species-rich predator assemblages would lead to a stronger reduction in arthropod damage because of higher predation rates, we have found that a higher species richness of birds and presumably also bats coincided with lower intensity of arthropod herbivory in the deciduous-mixed forest stands of the Hainich-Dün compared with the beech-dominated stands on the Schwäbische Alb. We can exclude to a large degree that regional differences in species richness and in abundance patterns of arthropods led to the documented regional differences in herbivory, because arthropod diversity was similar in the two study regions according to large-scale sampling in forest stands near the oak trees studied (Martin Gossner, unpublished data).

It is rather unlikely that other arthropod predators, parasites, and parasitoids, such as parasitic wasps, might have influenced our results, as the large mesh width of our nets allowed free access for them to both netted trees and controls. Hence, we assume that vertebrate predation is one of the major drivers leading to differences in intensity of arthropod herbivory between the two study sites.

Regional differences among the study sites might have contributed as well to the observed differences. Both study regions are about 300 km apart and differ in abiotic features such as topography and climate. The Schwäbische Alb is higher (500–900 m a.s.l.) than the Hainich-Dün (300–400 m a.s.l.) with lower annual mean temperatures (6–7°C vs. 6.5–7.5°C) and higher annual mean precipitation (700–1,000 mm vs. 750–800 mm). Hence, the more moderate climate of the Hainich-Dün might lead to higher herbivore abundance than the colder and wetter climate of the Schwäbische Alb. Contrary to this expectation we found higher intensity of herbivory on the Schwäbische Alb than in the Hainich-Dün. We assume that the observed differences are mainly due to differences in predator diversity as species richness and abundance of vertebrate predators were significantly higher in the Hainich-Dün than on the Schwäbische Alb and conclude that vertebrate predation (species richness and abundance of birds and bats) mainly influence herbivory.

As the canopy represents the major foraging habitat of foliage gleaning birds, a large number of insectivorous bird species feed on caterpillars, which constitute the most important herbivores in numbers and in species richness in temperate forest [4], [47]. In addition to the often rather specialized foliage-gleaning birds such as warblers, dietary preferences of some tree trunk gleaners such as *Dendrocopos major* (great spotted woodpecker) and *Sitta europaea* (European nuthatch) might further enhance herbivory control, as they have also been found to glean caterpillars off leaves [36]. Our interpretation that greater species richness of birds leads to a reduction of leaf-eating arthropods is further corroborated by the experimental study of Sanz [13] where the installation of nest-boxes for tits and flycatchers in a Pyrenean oak forest stand led to an increase in the number of insectivorous birds accompanied by a distinct reduction in the number of caterpillars in parallel with a reduction in leaf damage.

In addition to birds, foliage-gleaning insectivorous bat species are also likely to contribute to reduce herbivory in the canopy as they feed on herbivorous arthropods as well, mainly on caterpillars, moths and beetles [48]–[50]. In accordance with our observation of higher species richness of birds in the Hainich-Dün compared with the Schwäbische Alb, bat species richness and activity followed a similar pattern (Kirsten Jung, unpublished data). A large-scale monitoring study in both regions in the vicinity of the study oaks showed higher numbers of foliage-gleaning bat species in the Hainich-Dün (seven species) compared with the Schwäbische Alb (four species). Furthermore, the overall activity of foliage-gleaning bat species (number of passes per minute) was higher in the Hainich-Dün than on the Schwäbische Alb (Kirsten Jung, unpublished data).

To date, the effects of foliage-gleaning bats on the abundance of herbivorous arthropods have only been investigated in the tropics where the diversity of bat species and other groups in the food web are considerably higher [8], [10]. Kalka et al. [8] and Williams-Guillén et al. [10] have demonstrated a distinct impact of insectivorous birds and bats on the abundance of herbivorous arthropods and leaf damage. Moreover, the impact of foliage-gleaning bats on the reduction of leaf damage was even stronger than that of birds [8]. However, because of the lower numbers of foliage-gleaning bat species (≈ 7–10 species) in central Europe compared with that of insectivorous birds (≈ 70–80 species), we assume that birds contribute more to herbivore control than do bats, although this awaits further investigation. Because of logistic difficulties in conducting the study in the canopy, we could not differentiate between bird and bat predation in the canopy; it was impossible to remove and re-install the nets on a daily basis as carried out in the study of Kalka et al. [8] in the understorey and in the study of Williams-Guillén et al. [10] in agricultural areas.

In accordance with other studies, our results support the notion that higher species richness of vertebrate predators has a positive effect on herbivory control and ecosystem stability [7], [13], [18], [51]–[52]. Studies in tropical ecosystems have demonstrated that the species richness of insectivores, mainly birds, is the main driver controlling arthropods, because species richness of predators correlates with functional richness. Hence, higher species richness of predators increases the probability that highly efficient species are present [18]–[19], [53]. We suggest that in temperate systems, similar to the tropics, higher numbers of foliage-gleaning birds and bats are more efficient in herbivore reduction than species-poor predator assemblages (see also Philpott et al. [19]).

The protection of trees by birds and bats against excessive damage by herbivores might also directly affect the survival of individual leaves. As suggested by preliminary observations after our exclusion experiments, heavily damaged leaves might be shed earlier in the vegetation period than intact leaves (personal observations). Although trees were almost fully foliated at our last sampling season, our herbivory measurements probably were thus somewhat biased by early shed leaves. We thus propose that herbivory is also likely to affect the overall lifespan of leaves. Both factors leaf area and lifespan determine the primary production of deciduous trees [54]. Although plants can compensate loss of leaf area by production of new leaves, leaf re-growth incurs costs and increased risk that newly emerged and less protected leaves are more likely consumed by herbivores. The high production of new leaves might even temporarily lead to a population increase of herbivores [2].

Plants can protect themselves against herbivores and leaf damage by investing in secondary metabolites that lower leaf palatability for arthropods and/or aid as an olfactory cue to guide predators, parasitoids or foliage-gleaning insectivorous birds to infested leaves [55]. As shown by Mäntylä et al. [55], three volatile organic compounds ((E)-DMNT [(E)-4,8-dimethyl-1,3,7-nonatriene], β-ocimene, and linalool), that are involved in the attraction of parasitoids and predatory mites, were also positively correlated with increased predation rates of foliage-gleaning birds on *Betula pubescens* (mountain birches [55]). Overall, lower intensity of arthropod herbivory is likely to facilitate plant growth and reproduction as more resources are available to the plant [56]–[62].

To conclude, standardized quantitative and qualitative studies on the impact of birds and bats in the top-down regulation of herbivores at a broad geographical scale and under various diversity scenarios are indispensable for a better understanding of ecosystem functioning and deliver base-line data for the prediction of effects of environmental changes on ecosystem services relevant for forest primary productivity [12].

Our study shows that the top-down control of leaf damage strongly depends on predator diversity. Changes in species richness and abundance of birds and bats, e.g. through human induced changes in land use or climatic change, might have far-reaching consequences on ecosystem functioning and services, as a decrease in species richness of birds and bats are likely to lead to a steep increase in the number and abundance of arthropod herbivores. This is likely to profoundly influence intensity of leaf damage and, ultimately, fitness of trees and thus forest productivity [63]–[64].

Forest management practices with the aim of maintaining important ecosystem services should therefore consider conservation aspects, such as the preservation of structural heterogeneity, dead wood and ecologically valuable tree species, e.g. oaks, which provide cavities for the nesting and roosting of birds and bats. Moreover, additional conservation measures for birds and bats (e.g. maintenance of refugia habitats such as hedges and installation of nest-boxes) should be fostered in agricultural areas, where crops may also benefit from natural pest control, as has been demonstrated by Cleveland et al. [65] for *Tadarida brasiliensis* (Brazilian free-tailed bat).

Our study has revealed that bird and bat predation on arthropod herbivores significantly reduces leaf damage and biomass loss of the canopy in oak trees of the temperate zone. Profound changes in intensity and type of land use, including forest management, are accelerating reduction in animal and plant diversity in Europe. Therefore, in-depth studies on the functional roles of vertebrates are crucial if we are to predict reduction or loss of ecological function and services caused by decreasing diversity of bird and bat assemblages on the local and the regional level. A deeper understanding of the role of birds and bats in the top-down regulation of herbivory in the canopy of a temperate forest is a crucial step to providing baseline data for conservation decisions targeted at the maintenance of this important ecosystem service.

## References

[1] Hairston NG, Smith FE, Slobodkin LB (1960). Community structure, population control, and competition.. Am Nat.

[2] Utsumi S, Ohgushi T (2009). Community-wide impacts of herbivore-induced plant regrowth on arthropods in a multi-willow species system.. Oikos.

[3] Wright DM, Jordan GJ, Lee WG, Duncan RP, Forsyth DM (2010). Do leaves of plants on phosphorus-impoverished soils contain high concentrations of phenolic defence compounds?. Funct Ecol.

[4] Feeny PP (1970). Seasonal changes in oak leaf tannins and nutrients as a cause of spring feeding by winter moth caterpillars.. Ecology.

[5] Preszler RW, Boecklen WJ (1994). A three-trophic-level analysis of the effects of plant hybridization on a leaf-mining moth.. Oecologia.

[6] Forkner RE, Hunter MD (2000). What goes up must come down? Nutrient addition and predation pressure on oak herbivores.. Ecology.

[7] Van Bael SA, Philpott SM, Greenberg R, Bichier P, Barber NA (2008). Birds as predators in tropical agroforestry systems.. Ecology.

[8] Kalka MB, Smith AR, Kalko EKV (2008). Bats limit arthropods and herbivory in a tropical forest.. Science.

[9] Koh LP (2008). Birds defend oil palms from herbivorous insects.. Ecol Appl.

[10] Williams-Guillén K, Perfecto I, Vandermeer J (2008). Bats limit insects in a Neotropical agroforestry system.. Science.

[11] Gunnarsson B, Heyman E, Vowles T (2009). Bird predation effects on bush canopy arthropods in suburban forests.. Forest Ecol Manag.

[12] Marquis RJ, Whelan CJ (1994). Insectivorous birds increase growth of white oak through consumption of leaf-chewing insects.. Ecology.

[13] Sanz JJ (2001). Experimentally increased insectivorous bird density results in a reduction of caterpillar density and leaf damage to Pyrenean oak.. Ecol Res.

[14] Van Bael SA, Brawn JD (2005). The direct and indirect effects of insectivory by birds in two contrasting Neotropical forests.. Oecologia.

[15] Recher HF, Majer JD (2006). Effects of bird predation on canopy arthropods in wandoo *Eucalyptus wandoo* woodland.. Austral Ecol.

[16] Barber NA, Marquis RJ (2009). Spatial variation in top-down direct and indirect effects on white oak (*Quercus alba* L.).. Am Mid Nat.

[17] Mooney KA, Gruner DS, Barber NA, Van Bael SA, Philpott SM (2010). Interactions among predator and the cascading effects of vertebrate insectivores on arthropod communities and plants.. P Natl Acad Sci U S A.

[18] Perfecto I, Vandermeer JH, Bautista GL, Nunez GI, Greenberg R (2004). Greater predation in shaded coffee farms: the role of resident Neotropical birds.. Ecology.

[19] Philpott SM, Soong O, Lowenstein JH, Pulido AL, Lopez DT (2009). Functional richness and ecosystem services: bird predation on arthropods in tropical agroecosystems.. Ecol Appl.

[20] Gradwohl J, Greenberg R (1982). The effect of a single species of avian predator on the arthropods of aerial leaf litter.. Ecology.

[21] Altegrim O (1989). Exclusion of birds from bilberry stands: impact on insect larval density and damage to the bilberry.. Oecologia.

[22] Greenberg R, Bichier P, Cruz Angon A, MacVean C, Perez R (2000). The impact of avian insectivory on arthropods and leaf damage in some Guatemalan coffee plantations.. Ecology.

[23] Murakami M, Nakano S (2000). Species-specific bird functions in a forest-canopy food web.. Proc R Soc Lond B.

[24] Lichtenberg JS, Lichtenberg DA (2002). Weak trophic interactions among birds, insects and white oak saplings (*Quercus alba*).. Am Mid Nat.

[25] Mols CMM, Visser ME (2002). Great tits can reduce caterpillar damage in apple orchards.. J Appl Ecol.

[26] Bridgeland WT, Beier P, Kolb T, Whitham TG (2010). A conditional trophic cascade: Birds benefit faster growing trees with strong links between predators and plants.. Ecology.

[27] Schwenk WS, Strong AM, Sillett TS (2010). Effects of bird predation on arthropod abundance and tree growth across elevational gradient.. J Avian Biol.

[28] Gunnarsson B (1996). Bird predation and vegetation structure affecting spruce-living arthropods in a temperate forest.. J Anim Ecol.

[29] Gunnarsson B (2008). Bird predation on spiders: ecological mechanisms and evolutionary consequences.. J Arach.

[30] Gunnarsson B, Hake M, Hultegren S (2004). A functional relationship between species richness of spiders and lichens in spruce.. Biodivers Conserv.

[31] Millar CI, Stephenson NL, Stephens SL (2007). Climate change and forests of the future: managing in the face of uncertainty.. Ecol Appl.

[32] Feeny PP (1968). Effect of oak tannins on larval growth of the winter moth *Operophtera brumata*.. J Insect Physiol.

[33] Lanham JD, Keyser PD, Brose PH, Van Lear DH (2002). Oak regeneration using shelterwood-burn technique: management options and implications for songbird conservation in the southeastern United States.. Forest Ecol Manage.

[34] Southwood TRE, Wint GRW, Kennedy CEJ, Greenwood SR (2004). Seasonality, abundance, species richness and specificity of phytophagous guild of insects on oak (*Quercus*) canopies.. Eur J Entomol.

[35] Knoot TG, Schulte LA, Rickenbach M (2010). Oak conservation and restoration on private forestlands: negotiating a social-ecological landscape.. Environ Manage.

[36] Böhm SM, Kalko EKV (2009). Patterns of resource use in an assemblage of birds in the canopy of a temperate alluvial forest.. J Ornithol.

[37] Fischer M, Bossdorf O, Gockel S, Hänsel F, Hemp A (2010). Implementing large-scale and long-term functional biodiversity research: The Biodiversity Exploratories.. Basic Appl Ecol.

[38] Zuur AF, Ieno EN, Walker NJ, Saveliev AA, Smith GM (2009). Mixed effects models and extensions in ecology with R. Springer, Heidelberg.

[39] Bates D, Maechler M (2009). Lme4: Linear mixed-effects models using S4 classes. [WWW document].. http://cran.r-project.org/web/packages/lme4/index.html.

[40] Holmes RT, Schultz JC, Nothnagle P (1979). Bird predation on forest insects: an exclosure experiment.. Science.

[41] Gil-Tena A, Saura S, Brotons L (2007). Effects of forest composition and structure on bird species richness in a Mediterranean context: implications for forest ecosystem management.. Forest Ecol Manage.

[42] Kalcounis MC, Hobson KA, Brigham RM, Hecker KR (1999). Bat activity in the boreal forest: importance of stand type and vertical strate.. J Mammal.

[43] Coley PD (1986). Costs and benefits of defense by tannins in a Neotropical tree.. Oecologia.

[44] Strong DR, Sherry TW, Holmes RT (2000). Bird predation on herbivorous insects: indirect effects on sugar maple saplings.. Oecologia.

[45] Brossa R, Casals I, Pintó-Marijuan M, Fleck I (2009). Leaf flavonoid content in *Quercus ilex* L. resprouts and its seasonal variation.. Trees Struct Funct.

[46] Schnitzler H-U, Kalko EKV (2001). Echolocation by insect-eating bats.. Bioscience.

[47] Lill JT, Marquis RJ (2003). Ecosystem engineering by caterpillars increases insect herbivore diversity on white oak.. Ecology.

[48] Anderson ME, Racey PA (1991). Feeding behaviour of captive long-eared bats, *Plecotus auritus*.. Anim Behav.

[49] Swift SM, Racey PA (2002). Gleaning as a foraging strategy in 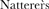 bat *Myotis nattereri*.. Behav Ecol Sociobiol.

[50] Goerlitz HR, ter Hofstede HM, Zeale MRK, Jones G, Holderied MW (2010). An aerial-hawking bat uses stealth echolocation to counter moth hearing.. Curr Biol.

[51] Paine RT (1980). Food webs: linkage, interaction strength and community infrastructure.. J Anim Ecol.

[52] McCann KS (2000). The diversity-stability debate.. Nature.

[53] Duffy JE, Cardinale BJ, France KE, McIntyre PB, Thébault E (2007). The functional role of biodiversity in ecosystems: incorporating trophic complexity.. Ecol Lett.

[54] Suwa T, Maherali H (2008). Influence of nutrient availability on the mechanisms of tolerance to herbivory in an annual grass, *Avena barbata* (Poaceae).. Am J Bot.

[55] Mäntylä E, Alessio GA, Blande JD, Heijari J, Holopainen JK (2008). From plants to birds: higher avian predation rates in trees responding to insect herbivory.. PLoS One.

[56] Coley PD, Bryant JP, Chapin FS (1985). Resource availability and plant antiherbivore defense.. Science.

[57] Moore R, Francis BJ (1991). Factors influencing herbivory by insects on oak trees in pure stands and paired mixtures.. J Appl Ecol.

[58] Wold EN, Marquis RJ (1997). Induced defence in white oak: Effects on herbivores and consequences for the plant.. Ecology.

[59] Terborgh J, Lopez L, Nunez VP, Rao M, Shahabuddin G (2001). Ecological meltdown in predator-free forest fragments.. Science.

[60] Hochwender CG, Sork VL, Marquis RJ (2003). Fitness Consequences of Herbivory on *Quercus alba*.. Am Midl Nat.

[61] Marquis RJ (2004). Herbivores rule.. Science.

[62] Vehviläinen H, Koricheva J, Ruohomäki K (2007). Tree species diversity influences herbivore abundance and damage: meta-analysis of long-term forest experiments.. Oecologia.

[63] Sekercioglu CH (2006). Increasing awareness of avian ecological function.. Trends Ecol Evol.

[64] Whelan CJ, Wenny DG, Marquis RJ (2008). Ecosystem services provided by birds.. Ann NY Acad Sci.

[65] Cleveland CJ, Betke M, Federico P, Frank JD, Hallam TG (2006). Economic value of the pest control service provided by Brazilian free-tailed bats in south-central Texas.. Front Ecol Environ.

